# Detecting tandem repeat variants in coding regions using code-adVNTR

**DOI:** 10.1016/j.isci.2022.104785

**Published:** 2022-07-19

**Authors:** Jonghun Park, Mehrdad Bakhtiari, Bernt Popp, Michael Wiesener, Vineet Bafna

**Affiliations:** 1 Department of Computer Science & Engineering, University of California, San Diego, La Jolla, CA 92093, USA; 2 Institute of Human Genetics, University Hospital Erlangen, Friedrich-Alexander University Erlangen-Nürnberg, Erlangen, Germany; 3 Institute of Human Genetics, University of Leipzig Hospitals and Clinics, Leipzig, Germany; 4 Department of Nephrology and Hypertension, University Hospital Erlangen, Friedrich-Alexander University Erlangen-Nürnberg, Erlangen, Germany

**Keywords:** Genetics, Genomics, Human genetics, Molecular genetics

## Abstract

The human genome contains more than one million tandem repeats (TRs), DNA sequences containing multiple approximate copies of a motif repeated contiguously. TRs account for significant genetic variation, with 50 + diseases attributed to changes in motif number. A few diseases have been to be caused by small indels in variable number tandem repeats (VNTRs) including poly-cystic kidney disease type 1 (MCKD1) and monogenic type 1 diabetes. However, small indels in VNTRs are largely unexplored mainly due to the long and complex structure of VNTRs with multiple motifs. We developed a method, code-adVNTR, that utilizes multi-motif hidden Markov models to detect both, motif count variation and small indels, within VNTRs. In simulated data, code-adVNTR outperformed GATK-HaplotypeCaller in calling small indels within large VNTRs. We used code-adVNTR to characterize coding VNTRs in the 1000 genomes data identifying many population-specific variants, and to reliably call *MUC1* mutations for MCKD1.

## Highlights

• Detection of coding variants in tandem repeats is confounded by ambiguous mapping
• Our method, code-adVNTR, detects variants in coding VNTRs using multi-motif HMMs
• code-adVNTR outperforms GATK-HaplotypeCaller on indel detection in tandem repeats
• A known frameshift variant within a VNTR in MUC1 gene was accurately detected

## Graphical abstract

**Figure:**
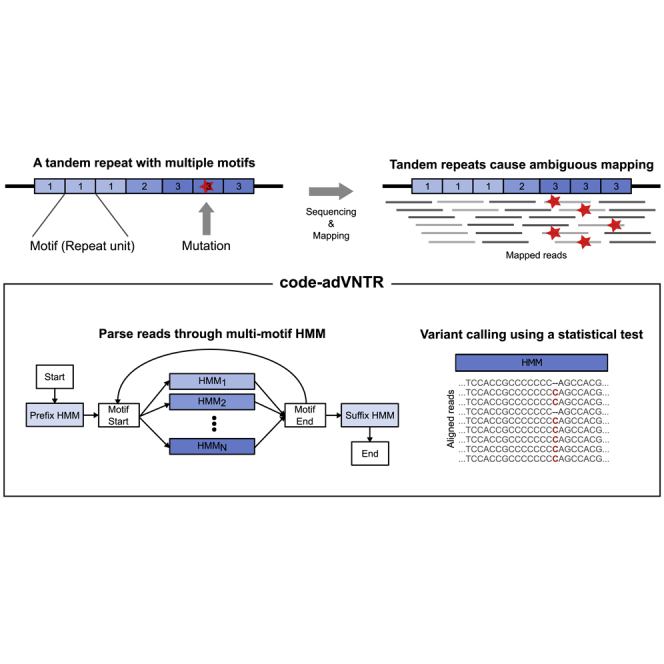

## Introduction

Tandem repeats (TRs) are characterized by DNA sequences containing multiple approximate copies of a motif repeated contiguously. Depending on the length of the motif, TRs are identified as “short” (STRs, motif length < 6bp), or variable number tandem repeats (VNTRs, motif length ≥ 6bp).

More than 50 diseases are known to be caused by repeat expansions (Hannan, 2021). Multiple computational tools have been devised to identify expansions, including methods based on sequence coverage (Mukamel et al., 2021; Dolzhenko et al., 2019), including through alignments to a pan-genome graph (Lu et al., 2021). Smaller changes in motif lengths cannot be detected solely by coverage, but are either detected directly through alignments as structural variants (Beyter et al., 2021) or can be explicitly detected by modeling the repeat structure as hidden Markov models (HMMs) (Bakhtiari et al., 2021). These smaller changes in VNTR motif counts often lie in non-coding regions and play a regulatory role mediating the expression of proximal genes (Bakhtiari et al., 2021). Even smaller indels and mutations within a VNTR are harder to genotype and available methods are limited (Mousavi et al., 2019; Bakhtiari et al., 2018; Dolzhenko et al., 2019, 2020).

Interestingly, an analysis using Tandem Repeats Finder (TRF) (Benson, 1999) suggests that ≥ 6,573 VNTRs lie within the coding region of a gene. Not surprisingly, most of these VNTRs are conserved in humans, and even small changes are likely to be correlated with a phenotype. However, the impact of coding VNTR variation on phenotypes has not been extensively studied, often because of the technical difficulties of genotyping. In a few cases, frameshift mutations in VNTRs have been found to be causal for monogenic diseases (Kirby et al., 2013; Ræder et al., 2006; Brookes, 2013). For example, autosomal dominant tubulointerstitial kidney disease (ADTKD) is known to be caused by a frameshift 1 bp insertion in the VNTR in *MUC1* gene, but this causal variant was not discovered until 2013 mainly due to the complex structure of the VNTR (Kirby et al., 2013). Specifically, *MUC1* has very long motifs (60 bp motifs, 20–125 copies) and is GC-rich making it difficult to obtain high coverage.

**Figure 1:**
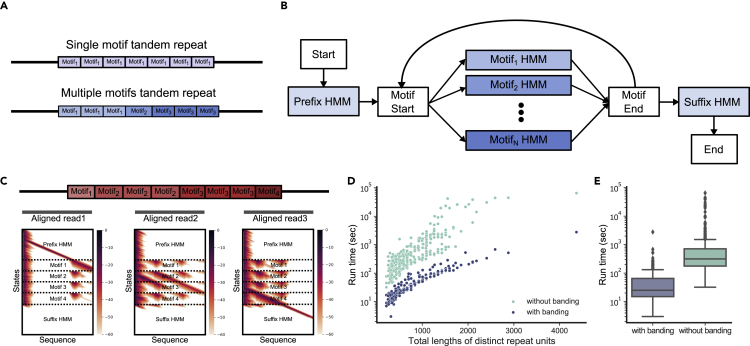
Small variant detection within VNTRs using code-adVNTR (A) An example of tandem repeats with single and multiple distinct motifs. The index of motif indicates distinct motifs, which are very similar but have variations in sequence composition. (B) The multi-motif HMM architecture of code-adVNTR. All HMMs are profile HMMs having insertion, deletion, and match states. The transition from Start node to all “match” nodes and “match” nodes to End node are allowed but not depicted for simplicity. (C) Three reads aligned to a VNTR region and the search space of Viterbi algorithm. In the search space, the white cells indicate the cells that were not explored during the process because there was no path to reach to the cell satisfying the score cut-off. (D) Distribution of run time as a function of the total lengths of distinct motifs with or without the banding algorithm. Note that the total lengths of distinct motifs are proportional to the number of states in HMMs. (E) Run time comparison of with or without the banding algorithm. Boxplots display the 25th, median, and 75th percentiles.

Long-read sequencing methods are now being utilized to identify mutations (Edge and Bansal, 2019), and also to genotype VNTRs using Nanopore (Beyter et al., 2021) and SMRT sequencing (Wenzel et al., 2018). However, the bulk of clinical pipelines and large population cohorts still utilize short-read sequencing to identify mutations due to cost and labor involved. Therefore, we focus on short-read identification of coding VNTR mutations. Two processes are at work in conferring VNTR variability. First, polymerase slippage may lead to changes in the total motif count; second, point mutations might scar and modify existing motifs. Therefore, many VNTRs are often composed of multiple distinct motifs (Course et al., 2021) (Figure 1A), each only approximately similar to the other. The VNTRs themselves can be denoted as “short” if they can be completely encompassed by short reads, and “long” otherwise. Small mutations in short VNTRs (but not entire motif count changes (Bakhtiari et al., 2018)) are well handled by existing mutation callers (McKenna et al., 2010).

Bakhtiari et al. developed adVNTR (Bakhtiari et al., 2018), which used hidden Markov models to parse a VNTR sequence into its constituent motifs. The HMM structure was also tolerant to small point mutations, including indels, allowing for accurate motif counts for short VNTRs with multiple motifs. However, adVNTR is not as successful for detecting indels within the motif, which is particularly relevant to coding VNTRs. Informally, one can consider the HMM procedure as creating a multiple alignment of the multiple repeat motifs. However, the genomic location of any repeat motif remains hidden and mutational changes relative to the reference cannot be registered (Figure S1).

To address these challenges, we present **code-adVNTR**, which focuses on (a) identifying entire motif insertions and deletions in short, coding VNTRs and, more importantly, (b) identifying small frameshifts and mutations in long, coding VNTRs.

## Results

### Coding VNTRs in the human genome

We focused on two sets of target VNTRs on the human genome: 2,254 *short coding VNTRs* (length ≤ 140 bp), where code-adVNTR was able to estimate the motif counts with short reads, and 364 *long coding VNTRs* (≥ 300 bp) where code-adVNTR could not detect motif counts reliably but could detect small indels that could not be easily identified by other mapping-based short-read variant callers.

### Overview of code-adVNTR

Code-adVNTR analyzes an input of short-read sequence data in two ways: it detects and reports (a) changes in motif count in a target list of short coding VNTRs and (b) small nucleotide variants within a target list of long coding VNTRs. Both analyses start by recruiting reads for each target VNTR, followed by variant identification using a custom profile HMM for the VNTR. For objective (a), code-adVNTR adopted adVNTR codebase (Bakhtiari et al., 2018, 2021). Very briefly, the profile HMM models the repeating motif and all of its variants (Figure S2). The read is parsed through the HMM while maintaining the motif count (the number of transitions through a special end-state). A subsequent Bayesian analysis calls the most likely diploid genotype as a pair of motif counts.

Code-adVNTR uses new algorithmic ideas to resolve objective (b): specifically, the new method can parse multiple profile-HMM motifs; second, it utilizes a novel algorithm to map reads to the HMM to restrict the search space and achieve orders of magnitude speed-up; and third, it utilizes a motif order alignment method to improve the accuracy of the HMM. These steps are outlined below, with additional detail in the methods section.

### Designing and parsing a multiple motif HMM

For detecting changes in motif counts for short coding, it is sufficient to parse using a single profile HMM (Figure S2). For long coding VNTRs, where the goal is to identify point mutations *within* VNTRs, code-adVNTR starts by running a single motif HMM on a reference VNTR sequence and parses and identifies all *distinct* (non-identical) motifs. It builds a separate profile HMM for each motif and for the flanking regions (prefix and suffix HMMs), and combines all HMMs into a single multi-motif HMM (Figure 1B).

### Restricting search space in Viterbi algorithm

Noting that multi-motif structure is also an HMM (though not a profile HMM), the Viterbi algorithm can be used unchanged to parse the read. However, a naive modeling of a VNTR with *u* motifs instead of a single motif causes a $u^{2}$ slow-down. To speed up computations, we use a “banding” idea to prevent excursion into states where the score is already too low (Figure 1C and Method details). Specifically, we utilize the fact that score worsens as we move away from Match state transitions. Once the score passes a threshold τ, that path will be guaranteed to lead to a non-optimal solution, and is prevented from further exploration. The score threshold τ is empirically and automatically computed based on the HMM and a user-defined parameter δ defined as the maximum number of indel transitions allowed in recruited reads (default δ = half of the motif length). We also note that the only way to complete a cycle in the state space graph is by transitioning from “Motif-End” to “Motif-Start”. We disallow degenerate cycles (that revisit a state without emitting any symbol) by topologically ordering the states and ignoring specific transitions (Method Details).

### Guidance from the reference sequence motif order

When a mutation in a motif makes it identical to another motif in the multi-motif HMM, it is matched to the second and would not be detected as a mutation. To differentiate true mutations from such internal sequence variations, we utilized reference sequences as follows: Each of the *u* motifs in a VNTR was given a distinct label *i* ranging from 1 to *u*. We built a look-up table of all possible motif-label orders based on the read length. For example, if the length of the motif was 30 bp, a read of 150 bp could span a maximum of five motifs, providing a sequence of five or fewer labels. Each read was parsed into its motif labels, and the look-up table was used along with a Smith-Waterman style local alignment algorithm to positionally align the read, allowing for up to one motif label mismatch. Thus, the read 1,2,2,1,3 is allowed to align to a longer reference label sequence 2,1,2,2,4,3,1,2. The alignment indicates that a mutation transformed motif 4 into motif 1. These alignments were used to *re-assign* substrings to individual motifs, while maintaining the overall output as collection of Viterbi paths for each motif.

### Variant detection within coding VNTRs

Code-adVNTR first recruits reads using various filtering steps (Bakhtiari et al., 2021) and maps the recruited reads to motifs using the profile HMMs. By decoding the most likely path (a series of states) using Viterbi algorithm, code-adVNTR counts the number of reads mapped to indel states for each motif. Given the number of indel transition, code-adVNTR calculates the likelihood of mutation as follows. Consider a read matching a motif *M* that occurs *u* times in the reference VNTR. For a heterozygous mutation, we expect to see 1/2u of the matched motifs to carry that mutation. For example, if u=1, then half of motifs aligning to motif *M* would carry the mutation in expectation. At a specific state covered by *d* reads, suppose *i* indel transitions were observed. Then, the likelihood of the observed mutation is given by $\text{Binomial}\left(d,i,\frac{1}{2u}\right).$ In contrast, the likelihood of seeing *i* indel transitions due to sequencing error is Binomial(d,i,ε), where ε is the probability of erroneous indel transition set to 0.01, a stringent per-nucleotide indel error rate based on the sequencing error rate in homopolymer regions of Illumina reads (Laehnemann et al., 2016). The log likelihood ratio

(Equation 1) $$-2\text{ln}\left(\frac{\text{Binomial}\left(d,i,\varepsilon \right)}{\text{Binomial}\left(d,i,\frac{1}{2u}\right)}\right),$$

is used for a statistical test, which follows a $\chi^{2}$ distribution. Code-adVNTR reports the mutation if the nominal p value is lower than 0.001.

### Benchmarking code-adVNTR

We tested the reduction in search space with code-adVNTR using simulated reads at 30× coverage from 364 long coding VNTRs (Method Details). Notably, code-adVNTR explored only ∼ 20% of the original search space on average. The run time was improved by 19-fold on average (3–191-fold). The median run time was 25 s with our “banding” algorithm. (Figure 1D). We also tested the run time in high-coverage (30×) 1000 Genomes Project (1KGP) data (1000 Genomes Project Consortium et al., 2015). On whole genome sequencing data, we used the adVNTR codebase (Bakhtiari et al., 2018) to assign each read to a specific VNTR location (or discard it), and genotyped each set of reads assigned to each VNTR. Code-adVNTR genotyped 2,237 VNTRs in 179 min per sample on a single core of Dual Intel Xeon Skylake 6132 2.60GHz CPU. For long coding VNTRs, code-adVNTR took 7.35 h per sample to scan all 364 VNTRs using Algorithm 1 and reference label alignments.

Algorithm 1 **Banding in code-adVNTR**

**Input:** {S,T,V,e,τ}
**Output:** Score of the best parse
$Q_{0}$.enQueue(start-state)
**for** 0≤j≤m **do**
**while** $Q_{j}\neq \left\{\right\}$ **do**
    $p\leftarrow Q_{j}$.de-Queue()
**if** j<m **then**
      **for** non-silent *q* s.t. T[p,q]>0 **do**
        $\text{tmp}\leftarrow V\left[p,j\right]+\text{log}T\left[p,q\right]+\text{log}e_{q}\left[r_{j+1}\right]$
        **if** tmp ≥max{τ,V[q,j+1]} **then**
          V[q,j+1]←tmp
          $Q_{j+1}$.enQueue(*q*)
    **for** silent *q* s.t. T[p,q]>0 **do**
      tmp←V[p,j]+logT[p,q]
      **if** tmp ≥max{τ,V[q,j]} **then**
        V[q,j]←tmp
        $Q_{j}$.enQueue(*q*)
**if** $V\left[\text{end-state)},m\right]>\tau$ **then return** $V\left[\text{end-state)},m\right]$
**else return** “no alignment”

### Code-adVNTR outperforms GATK in VNTR indel detection

**Figure 2:**
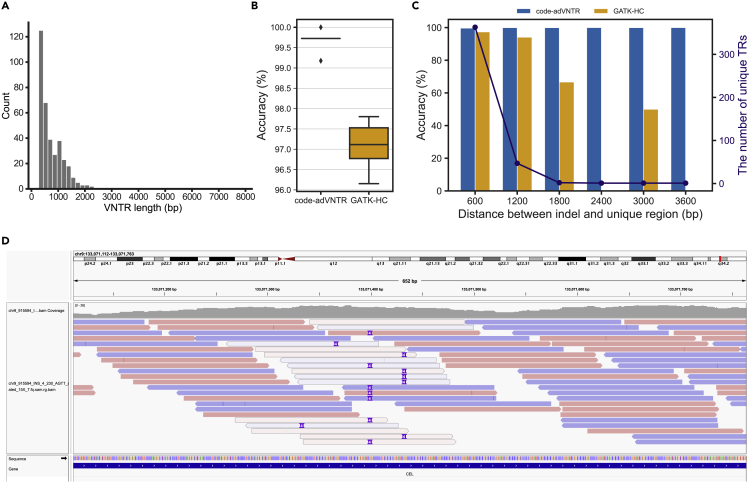
Performance comparison of variant detection in long VNTRs using simulated data (A) The length distribution of 364 long coding VNTRs (≥ 300 bp). (B) Average accuracy distributions of random indel detection for 364 coding VNTR in 10 simulations. Boxplots display the 25th, median, and 75th percentiles. (C) Accuracy comparison of the indel detection in 364 coding VNTRs as a function of distance from indel to unique region. The bar plot (left Y axis) shows the accuracy, and the line plot (right Y axis) shows the number of unique tandem repeats in certain range of distance. (D) Ambiguous mapping of short reads in a long coding VNTR within *CEL* gene. Note that the reads containing the simulated insertion (purple vertical bars) are mapped to multiple positions with low mapping scores (light gray).

The accuracy of calling motif count changes in short VNTRs using adVNTR has previously been documented (Bakhtiari et al., 2018, 2021), and we mimic the procedure for short coding VNTRs. Therefore, we focused here on testing code-adVNTR’s accuracy in calling indels within 364 long coding VNTRs (Figure 2A) using simulated reads and compared accuracy against GATK-HaplotypeCaller (GATK-HC) (McKenna et al., 2010). For each target VNTR locus, we modified the reference sequence by inserting or deleting a random number $\left(<\frac{\text{length}(\text{motif})}{2}\right)$ of nucleotides at a random position within the VNTR and simulated reads from the mutation. We repeated the experiment 10 times.

We measured accuracy for each experiment using the fraction of indels called correctly. Code-adVNTR correctly identified most of the cases (accuracy of 99.70%), in contrast with GATK-HC which showed an accuracy of 97.16% on average (Figure 2B). As code-adVNTR is built on top of adVNTR, we also tested adVNTR (Bakhtiari et al., 2018) on the same data. AdVNTR is not designed for mutation detection and only achieved an accuracy of 71.46%, suggesting that the use of multiple motifs is critical. To evaluate the contribution of the label alignment procedure to accuracy, we also tested code-adVNTR without the guidance of motif order (Method Details). Without the label alignment, code-adVNTR missed three additional cases out of 3,640.

As the difficulty of identifying mutation is related to the difficulty of aligning/mapping repetitive sequences, the position of the mutation is crucial. If a mutation is located near flanking unique regions, detecting such mutations becomes as easy as other mutations in non-repeat regions as reads can be mapped unambiguously. Figure 2C shows the accuracy stratified by the distance between the simulated indel and the unique region. The accuracy of GATK-HC was dropped when the indel is far from the flanking regions due to the ambiguous mappings (Figure 2D).

We additionally investigated performance on VNTRs where mutations have previously been found to be causal for phenotypes. These include *MUC1*, where a frameshift is causal for autosomal dominant tubulointerstitial kidney disease (ADTKD) (Kirby et al., 2013); *CEL*, where deletion mutations in the VNTR have been implicated in mature onset diabetes and other pancreatic disorders (Torsvik et al., 2010; Gravdal et al., 2021); and *PER3*, where VNTR length has been associated with diurnal preference and sleep homeostasis, and also with age of onset of bipolar disorder type I (Benedetti et al., 2008). We tested for prediction accuracy using simulations with randomly inserted mutations. The detection accuracy of GATK-HC was relatively low for *PER3* (80% accuracy), *MUC1* (80%), and *CEL* (50%), while code-adVNTR achieved 100% accuracy on these VNTRs.

### Long-read validation

To validate code-adVNTR’s calls using long reads, we tested code-adVNTR on the genomic sample NA12878 for which both short and long reads are available. We applied code-adVNTR to the 364 long coding VNTRs using only short-read data. Code-adVNTR identified three single base deletions in two VNTR loci in *ZNF662* (motif length 84, total VNTR length 588) and *ZNF717* (motif length 84, total VNTR length 1,845) genes. We confirmed the three deletions by reviewing the alignment of long reads, and all three showed up as heterozygous deletions confirming that code-adVNTR can detect heterozygous indels accurately (Figure S3). Code-adVNTR tolerates up to four mismatches per motif by default. In hyper-variable regions that contain more than four SNPs within a single motif, code-adVNTR will not make an indel call. Indeed, the hyper-variable VNTR region in *ZNF717* contained more than four common SNPs in the motif, and code-adVNTR specifically missed three indels. We next checked systematically for such hyper-variable regions using dbSNP data, and found that 27 coding VNTRs out of 364 contain motifs with four or more SNPs. The motifs span 0.97% of the target VNTR regions. Detecting mutations in these regions could be challenging for code-adVNTR if an individual has the non-reference allele in more than four SNPs in a moif.

### Variants within coding VNTRs in 1000 Genomes Project Datasets

We investigated variants in two sets of coding VNTRs: 2,254 short coding VNTRs (≤ 140 bp), where code-adVNTR is able to estimate the repeat counts with short reads, and 364 longer coding VNTRs (≥ 300 bp) for small indel variants. Consistent with the strong conservation of coding sequence, 1,989 (88.2%) of the 2,254 short coding TRs did not show any variation in 2,504 individuals.

Before analyzing the short coding VNTRs that showed polymorphisms, we filtered out 126 VNTRs that failed the Hardy–Weinberg equilibrium (HWE) test with p value cutoff of 0.05. Significant violation of HWE could be attributed largely to two reasons—long VNTRs and ambiguous boundaries. Rejected VNTRs had significantly longer lengths (median length of 75 bp) compared to the VNTRs that passed HWE test (median length of 54) (Figure S4A). We also observed that VNTRs that failed HWE test had higher similarity with the flanking regions (Figure S4B) making it challenging to distinguish between a motif and the flanking regions.

**Figure 3:**
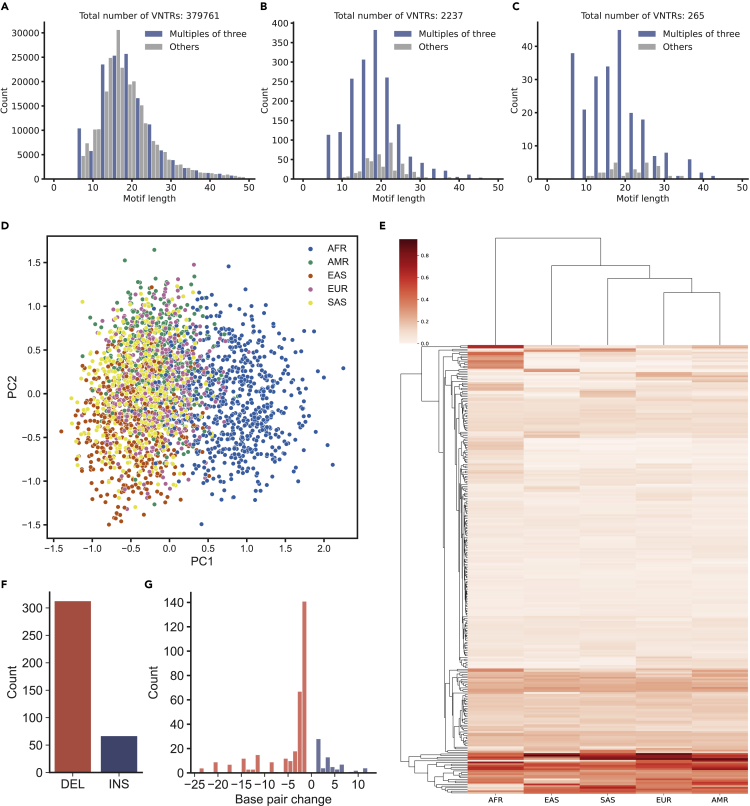
Coding VNTR variants in 1000 Genomes Project data (A and C) Motif length distribution of (A) VNTRs detected by Tandem Repeat Finder, (B) VNTRs in coding regions, and (C) polymorphic VNTRs in coding regions. (D) Principal component analysis (PCA) of polymorphic VNTRs. PC1 separated African super population from others. East Asian population (EAS) was weakly separated from others along with PC1 and PC2. (E) Clustered heatmap of normalized entropy matrix of polymorphic VNTR alleles. For each VNTR, normalized entropy was calculated by considering each allele (motif count). A Cluster map shows that the African super-population is distant from other super populations, which is consistent with the PCA analysis. (F) Small variant type (insertion or deletion) and the count detected in 364 coding VNTRs in 1000 Genomes Project Data. (G) The distribution of base pair changes of 380 indels detected by code-adVNTR.

After the filtering using HWE test, 265 polymorphic VNTRs remained. For these VNTRs, we checked if the consensus motif lengths were multiples of three (*zero-mod3 motifs*) because changes in motif count may result in frameshifts in coding regions. Notably, when we look at all TRs reported by TRF (Benson, 1999), only 36.36% were zero modulo 3 (Figure 3A). In contrast, 79.09% of the 2,254 short coding TRs (Figure 3B), and 87.54% of the polymorphic VNTRs were zero modulo 3 (Figure 3C), implying the motif count changes were primarily in-frame variants.

We asked if the polymorphisms in coding VNTR were sub-population specific. A principal component analysis on all individuals using their allelic values (Method Details), separated out the African samples using PC1 (AFR in Figure 3D). The other populations were not separated except for a weak separation of East Asians along PC2. We additionally measured the entropy of the allelic distribution of each VNTR in each population and found once again (Figure 3E), that a subset of VNTRs showed high entropy in AFR relative to other populations, while other high-entropy VNTRs were uniformly distributed among all populations. Together, our results suggest generally low levels of VNTR allelic diversity (high conservation) as expected in coding VNTRs and also a reduction of allelic diversity once humans moved out of Africa.

In the 364 long coding VNTRs, code-adVNTR identified 380 small indel variants in 180 VNTRs. Of those, 313 were deletions and 67 were insertions (Figure 3F). For both insertions and deletions, single base pair variant were the most frequent (Figure 3G). Interestingly, only 161 of 380 indels (42.37%) were multiples of three indicating that a large fraction of these mutations changed the coding frame. We compared the indels with the variant calls generated by Centers for Common Disease Genomics (Byrska-Bishop et al., 2021) (STAR Methods), which used straight mapping-based methods to call variants. 349 of the 380 variants had been reported earlier, but 31 indels called by code-adVNTR were novel. Most of these variants are rare (Table S4), but 8 (2 insertions, six deletions) were found in ≥ 10 individuals each, and many of these include genes with known Mendelian phenotypes.

As one example, we found a 13 bp deletion in exon four of the neurofilament heavy (*NEFH*) gene in 143 individuals (Figure S5A). The *NEFH* gene has been implicated in amyotrophic lateral sclerosis (ALS), a progressive motor neuron degeneration that leads to paralysis and death (Al-Chalabi et al., 1999). The report suggests that deletions in a repeating protein motif (XKSPYK), where X and Y are variable amino acids are associated with ALS. The motif corresponded exactly to the VNTR.

In 13 individuals, we found a 9 bp deletion in a VNTR located in exon five of the formin 2 (*FMN2*) gene (Figure S5B), belonging to a family of actin cytoskeleton genes. Heterozygous deletions of the distal exons (Almuqbil et al., 2013) as well as truncating mutations (Law et al., 2014) in the gene have been associated with autosomal-recessive intellectual disability.

Mutations in Keratin-9 have been associated with epidermolytic palmoplantar keratoderma (EPPK, OMIM 144200), an autosomal dominant inherited disease (Li et al., 2019). We identified a 13 bp deletion in the exon seven VNTR of *KRT-9* in 20 individuals (Figure S5C).

Finally, mutations in the retinitis pigmentosa-1-like-1 (*RP1L1*) gene are known to be causal for autosomal dominant occult macular dystrophy (OCMD) (Zobor et al., 2018). We identified a 24 bp deletion in exon four of *RP1L1* in 10 individuals (Figure S5D).

### Detecting frameshift variants in MUC1 VNTR

**Figure 4:**
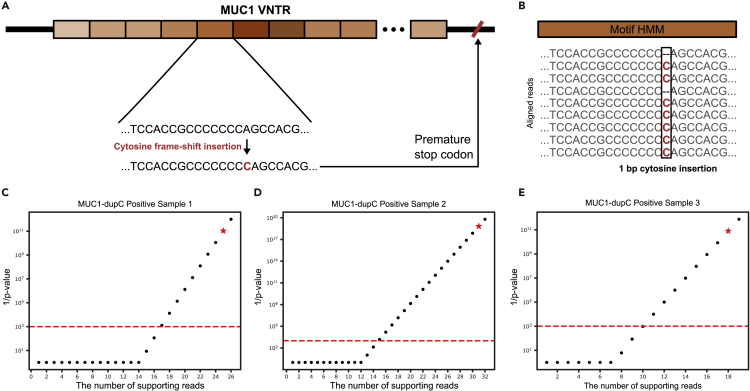
Pathogenic frameshift variants detection in MUC1 VNTR (A) A VNTR within MUC1 gene and the mechanism of known pathogenic cytosine insertion (MUC1-dupC) causing a premature stop codon resulting in autosomal dominant tubulointerstitial kidney diseases. (B) An example of reads aligned to a motif and variant detection in code-adVNTR. (C–E) The inverse of p value as a function of the number of supporting reads of three MUC1-dupC positive samples. Black dots show the trend, and red stars indicate the number of supporting reads and the corresponding p value observed in the MUC1-dupC-positive samples. Red dotted line shows the p value cutoff.

Autosomal dominant tubulointerstitial kidney disease (ADTKD) is a collection of rare kidney disorders, associated with terminal loss of kidney function. They are known to be caused by mutations in several genes, one of which is a single cytosine frameshift insertion at a VNTR within *MUC1* gene. The underlying mechanism was revealed only in 2013 (Kirby et al., 2013) because of the complex structure of the VNTR composed of 25–120 repeats of approximately 60 bp motifs in a GC-rich region, making it hard to detect using conventional technology (Figure 4A).

Long reads have been used to detect the variant, but many clinical pipelines still use short-read sequencing due to labor and cost (Wenzel et al., 2018). Recent methods designed a panel (SNaPshot) to enrich coverage of short reads, but the alignments had to be inspected manually to actually identify the variant (Ekici et al., 2014).

We applied code-adVNTR on three individuals previously identified as MUC1-dupC positives (carrying a cytosine frameshift insertion) using SNaPshot technology (Ekici et al., 2014), and also three MUC1-dupC-negative individuals for comparison. Code-adVNTR successfully identified all three MUC1-dupC-positive individuals using multi-motif HMM (Figure 4B) with enough supporting reads (Figures 4C–4E) and did not find any variants in the negative samples. Next, we applied code-adVNTR to 271 DNA samples from German Chronic Kidney Disease cohort (Popp et al., 2021) and cross-checked with the SNaPshot protocol (Ekici et al., 2014). All 271 samples were classified as MUC1-dupC negative by both code-adVNTR and SNaPshot.

## Discussion

Genetic variation in tandem repeats is challenging to identify. Notably, the mode of variation itself is complicated. For example, the number of motifs can sometimes expand dramatically. However, in other cases, the number of motifs may change by one or two and still cause a change in phenotype. Finally, single nucleotide variants might occur within motif regions.

Methods are being developed to identify these multiple types of TR variations modes. For example, changes in sequence coverage as also changes in k-mer count frequency can be used to detect large expansions (Mukamel et al., 2021; Lu et al., 2021). Those methods can work with short reads as long as the expansions are large enough to cause a significant change in coverage or k-mer counts. For more subtle changes in the number of VNTR motifs, a parsing or decomposition of the motifs can be used to detect those changes (Bakhtiari et al., 2018), and these subtle changes also may have phenotypic consequences. They may affect changes in the regulatory circuitry and the expression of proximal genes (Bakhtiari et al., 2021). These methods, however, work best when the read spans the VNTR, and long reads may be required for longer VNTRs. Mutations in VNTRs are particularly hard to detect. For example, if two inexact motifs A and B are both present in a VNTR, a mutation in A might make it identical to B.

Here, we focus on variations in coding VNTRs. Coding VNTRs represent a slightly easier case as they are less variable than other VNTRs. We focus specifically on identifying insertions and deletions of small numbers of nucleotides as well as changes in motif counts that would change the coding sequence. These mutations are hard for traditional pipelines, and also cannot be genotyped using coverage-based estimates of TR variation because of smaller levels of expansion or contraction of motifs.

We provide customized multi-motif HMM-based genotyping of VNTRs. Our methods are optimized using a banded Viterbi method, a label alignment after an initial motif alignment, and statistical calculation to distinguish mutations from sequencing errors. Our methods work for motif count variation in small VNTRs and small indels/mutations in long VNTRs. Motif count changes in long VNTRs will likely be detected using long-read technologies, although coverage-based methods might also detect large expansions of motif counts. Recent work aimed at isolation of specific target loci followed by long-read sequencing can help in that effort. Future research will focus utilizing long reads to improve anchoring to the correct locations.

Our results on the coding VNTRs in 2504 individuals suggest that most coding region repeats are stable; however, there is greater diversity in the African populations relative to other continents. Interestingly, we found many instances of VNTR coding region changes in multiple individuals and where the gene was previously associated with a Mendelian phenotype. Overall, our results suggest a rationale for including VNTR mutation analyses as part of Mendelian disease pipelines.

### Limitations of the study

Code-adVNTR also has a number of limitations, which will be addressed in future work. Notably, code-adVNTR can work with VNTRs that are longer than the read length. However, in identifying indels, code-adVNTR does not pin-point the exact location of the detected variants, but only its relative position in a specific motif. This makes it challenging to validate experimentally, as also compared with existing variant calls. Nevertheless, code-adVNTR takes an important step by identifying mutations that causes a frameshift in coding sequence. Second, this is not a methodological problem but rather, a limitation of short reads. In future work, we will extend code-adVNTR to include PacBio HiFi reads as well as other sequencing methods.

Second, parsing reads through a multiple motif HMMs could be slow when there are too many unique motifs because code-adVNTR builds HMM for each unique motif. The number of states in the HMMs is proportional to the sum of the lengths of the distinct motifs. Thus, both the length of the motif and number of distinct motifs are crucial. As one example, the VNTR in ZNF662 gene is composed of 22 distinct motifs where the lengths are 84 bp for each. The processing time for ZNF662 was 308 times slower than the times for another VNTR with a single motif of 6 bp.

Finally, and as suggested by the name, code-adVNTR is not tested for non-coding VNTRs, which could be hyper-variable, relative to coding VNTRs. Specifically, if a motif encodes multiple SNPs, identifying indels while accounting for the natural variation becomes difficult. The default number of mismatches tolerated per motif by code-adVNTR is currently 4. Similarly, detection of indels in the VNTR has not been systematically tested for non-coding VNTRs or VNTRs where indels are accompanied by additional large expansion in the number of motifs. Again, with the larger context provided by longer reads, these constraints can be relaxed, and this will be tested in future work. In the meantime, code-adVNTR has high utility for identification of indels and small motif counts in coding VNTRs.

## STAR★Methods

### Key resources table

**Table:** 

REAGENT or RESOURCE	SOURCE	IDENTIFIER
**Deposited data**		

1000 Genomes Project	1000 Genomes Project Consortium et al., 2015; Byrska-Bishop, Marta et al. (2021)	https://www.internationalgenome.org/data-portal/data-collection/30x-grch38
1000 Genomes Project variant calls	Byrska-Bishop, Marta et al. (2021)	http://ftp.1000genomes.ebi.ac.uk/vol1/ftp/data_collections/1000G_2504_high_coverage/working/20190425_NYGC_GATK/
NA12878 PacBio BAM file	Genome in a Bottle, PacBio	https://ftp-trace.ncbi.nlm.nih.gov/ReferenceSamples/giab/data/NA12878/PacBio_SequelII_CCS_11kb/HG001_GRCh38/

**Software and algorithms**		

code-adVNTR	This paper	https://github.com/mehrdadbakhtiari/adVNTR
GATK	McKenna et al. (2010)	https://gatk.broadinstitute.org/hc/en-us
ART	Huang, Weichun et al. (2012)	https://www.niehs.nih.gov/research/resources/software/biostatistics/art/index.cfm
IGV	Robinson et al., 2011	https://software.broadinstitute.org/software/igv/

### Resource availability

#### Lead contact

Further information and requests for resources and reagents should be directed to and will be fulfilled by the lead contact Jonghun Park (jop002@eng.ucsd.edu).

#### Materials availability

This study did not generate new unique reagents.

### Method details

#### Target coding VNTRs

We started with 693,770 variable number tandem repeats (VNTRs) found by Tandem Repeat Finder (TRF). We focused on VNTRs with 6 ≤ pattern length ≤ 100, repeat count ≥ 2, total length ≤ 30,000 to exclude short tandem repeats and very long tandem repeats (satellites). In this study, we focused on VNTRs lying within coding regions of genes using refseq gene coordinates because any mutations in those regions are likely to be functionally associated with the corresponding protein product.

To investigate variations in repeat counts, we focused on *short-coding-VNTRs* with total length ≤ 140 bp. We filtered out VNTRs if a VNTR has a similar sequence with another TR by comparing the pattern and flanking regions. To select non-overlapping TRs, we sorted the TRs by the end positions and greedily selected one that ends first. A total of 2237 TRs remained (Table S1).

To investigate small variants within motifs in coding VNTRs, we selected VNTRs with total length ≥ 300 bp (*long-coding-VNTRs*), which can not be easily detected by other tools due to multiple mapping problem with short reads. After applying the same filtering methods used to select *short-coding-VNTRs*, we additionally added three disease-associated VNTR loci with in *PER*, *MUC1*, and *GP1BA* genes. Overall, 364 TR loci (343 unique genes) are selected in our target loci list (Table S3).

#### Basics of the adVNTR HMM

Recall that any HMM *M* is defined by a 5-tuple M={S,Σ,T,e,π}. Here, Σ={A,C,G,T} denotes the emitted symbols, *S* denotes the set of states, T,e denote the transition and emission probabilities, respectively, and π denotes the initial probability distribution on the set of states. In the single motif profile HMM used by adVNTR (Figure S2), we always start at a begin state, or $\pi_{q}=1$ when q=BEGIN, and $\pi_{q}=0$ otherwise. The motif itself is represented by a sequence of Match states, with additional Insert states and Delete states. At the end of the motif, a transition is allowed back to the beginning of the motif as well as to the END state. In each step, the HMM emits a symbol (unless it is at a non-emitting state), and then transitions to a new state.

The Viterbi algorithm is used to identify the most likely sequence of states traversed while emitting the DNA sequence provided as input. Specifically, let V[q,j] denote the highest (log) probability of emitting the first *j* letters of the sequence $r_{1},r_{2},\ldots r_{m}$ (length *m*) and ending in state q∈S of an HMM with state space *S* (|S|=n). Then for all 1≤j≤m, and all q∈S

(Equation 2) $$V\left[q,j\right]=\left\{ \begin{array}{cc} \underset{p\in S}{\text{max}}\left\{V\left[p,j\right]+\text{log}T\left[p,q\right]\right\} & q\;\text{is a ‘silent’ state }\\ \underset{p\in S}{\text{max}}\left\{V\left[p,j-1\right]+\log\;T\left[p,q\right]+\log\;e_{q}\left[r_{j}\right]\right\} & \text{otherwise}, \end{array}\right.$$

where ‘silent’ states refer to the states that do not emit any letters, and T[p,q] denotes the transition probability from state *p* to *q* and $e_{q}\left[r_{j}\right]$ denotes the emission probability of $r_{j}$ in state *q*. Each iteration takes O(n) steps for a total time of $O\left(n^{2}m\right)$ steps required to parse.

#### Designing multiple motif HMM

code-adVNTR executes the following steps for constructing he multi-motif HMMs: (1) use the single motif HMM based adVNTR to parse the reference VNTR sequence into specific and distinct motifs. (2) Builds a separate profile HMM for each motif and for the flanking regions (prefix and suffix HMMs). and, (3) combine all HMMs into a single multi-motif HMM (Figure 1B). The HMM for each motif is a standard profile HMM. As mutations, and specifically indels, are much rarer in coding sequence, the transition probabilities were set using an empirical analysis of known VNTRs. Specifically, the transitions from the start state to *m* motifs were set to 1/m. Within the profile HMM for each motif, the transition from Match to Match state was set to 0.9975. Similarly, the emission probability of the reference residue was set to 0.996. The emissions of non-reference residues, and transitions to insert or delete states were set to 0.00125. As most motifs have only a small probability of a non-reference transition (due to a coding SNV or sequencing error), repeated traversal of the same non-reference transition during parsing is a signal for a coding indel.

#### Restricting search space in Viterbi algorithm

Noting that multi-motif structure is also an HMM (though not a profile HMM), the Viterbi algorithm can be used unchanged to parse the read. The number of states *n* is largely determined by the number of motifs *u* in the model and the length *w* of each motif (n∝uw). Thus, a naive modeling of a VNTR with *u* motifs instead of one changes the parsing time for a sequence of length *m* from $O\left(w^{2}m\right)$ to $O\left(u^{2}w^{2}m\right)$, causing a $u^{2}$ slow-down.

To speed up computations, we use a ‘banding’ idea to prevent excursion into states where the score is already too low (Figure 1C). The score threshold τ is empirically and automatically computed based on the HMM and a user-defined parameter δ defined as the maximum number of indel transitions allowed in recruited reads (default δ = half of the motif length). We also note that the only way to complete a cycle in the state space graph is by transitioning from ‘Motif-End’ to ‘Motif-Start’. We disallow degenerate cycles (that revisit a state without emitting any symbol) by topologically ordering the states while ignoring that transition. We maintain $Q_{j}$ as a *queue* of states *q* that remain active after reading the first *j* symbols. Then, $Q_{0}=\text{start}-\text{state}$, and $Q_{j}=\{q:V\left[q,j\right]>\tau$}). In Algorithm 1, we iterate over all *j* and repeat the steps outlined until $Q_{j}$ is empty.

The algorithm works by maintaining the following invariants for all 1≤j<m: (a) State *p* is added to $Q_{j}$ only if V[p,j]≥τ; (b) when state *p* is removed from $Q_{j}$ for the last time, then for all non-silent *q*, $\text{max}\left\{\tau ,V\left[q,j+1\right]\right\}\geq V\left[p,j\right]+\text{log}T\left[p,q\right]+\text{log}e_{q}\left[r_{j+1}\right]$; and for all silent *q*, max{τ,V[q,j]}≥V[p,j]+logT[p,q]. Invariant (a) is direct from the algorithm. For invariant (b), let $t\left[p,q\right]=V\left[p,j\right]+\text{log}T\left[p,q\right]+\text{log}e_{q}\left[r_{j+1}\right]$ for some non-silent *q*. The algorithm ensures that if t[p,q]≥τ, then V[q,j+1]≥t[p,q]. A similar argument holds for silent *q*.

We can use these invariants to assert that when $Q_{j}$ is emptied, then V[p,j] holds the maximum achievable score for emitting the first *j* symbols and ending in state *p* so long as the maximum score exceeds τ. Suppose the assertion is not true at *j*, and there exists V[p,j] which has the highest score among all states that failed to achieve their maximum value. The maximum value either equaled $V_{1}=V\left[q,j-1\right]+\text{log}T\left[q,p\right]+\text{log}e_{p}\left[r_{j}\right]$ for some *q*, or $V_{2}=V\left[q,j\right]+\text{log}T\left[q,p\right]$ for some *q*. In the first case, state *q* had been removed from $Q_{j-1}$ for the last time, and using assertion *b*, $V\left[p,j\right]\geq V_{1}$. In the second case, we know that V[q,j]’s score is higher than the maximum score achievable by V[p,j] and it is therefore correct. Then, by invariant (b), $V\left[p,j\right]\geq V_{2}$ when *q* was removed from $Q_{j}$ for the last time. We used backtracking to parse each readthrough the multi-motif HMM and used the Viterbi path to partition each read and to align each sub-string in the partition to a flanking sequence or a motif.

#### Guidance from the reference sequence motif order

When a mutation in a motif makes it identical to another motif in the multi-motif HMM, it is matched to the second and would not be detected as a mutation. To differentiate true mutations from such internal sequence variations, we utilized reference sequences as follows: Each of the *u* motifs in a VNTR was given a distinct label *i* ranging from 1 to *u*. We built a look-up table of all possible motif-label orders based on the read length. For example, if the length of the motif was 30 bp, a read of 150 bp could span a maximum of five motifs, providing a sequence of 5 or fewer labels. Each read was parsed into its motif labels, and the look-up table was used along with a Smith-Waterman style local alignment algorithm to positionally align the read, allowing for up to one motif label mismatch. Thus, the read 1,2,2,1,3 is allowed to align to a longer reference label sequence 2,1,2,2,4,3,1,2. The alignment indicates that a mutation transformed motif 4 into motif 1. These alignments were used to re-assign substrings to individual motifs, while maintaining the overall output as collection of Viterbi paths for each motif.

#### Performance comparison using simulated reads

To evaluate the performance of indel detection in VNTRs, we generated 10 whole genome sequencing data for each target locus. We simulated heterozygous mutations by putting an indel mutation with a random size $\left(<\frac{\text{length}(\text{motif})}{2}\right)$ at a random position in each TR locus. Then, we simulated reads (30× coverage) from the human reference genome (GRCh38) using ART (Huang et al., 2012) with Illumina HiSeq 2500 error profile.

We compared the performance of indel detection of code-adVNTR with GATK4 Haplo-type-Caller. We measured accuracy for each simulation, calculated by the number of correctly identified VNTRs divided by the total number of target VNTRs. We regarded a call as true when a tool found any mutations in the VNTR region because it is not always possible to locate the exact position of a variant in repeated sequences, which can not be spanned by a read. Although code-adVNTR does not localize observed mutation, in many cases such as known pathogenic mutations or diagnostic tests, it is sufficient to know the existence of the mutation in pathogenic VNTRs, and the results can be used to prioritize candidates for the further experiments to confirm the cases. We also measured running time of code-adVNTR by running them with default parameters on a single core of Intel Xeon CPU E5-2643 v2 3.50GHz CPU.

#### Identifying coding VNTR variants in 1000 Genomes Project data

Code-adVNTR has two modes: estimating motif count, and detecting variants within motifs in VNTRs. To investigate coding VNTR variants, we focused on the two different sets of target coding VNTRs (short- and long-coding VNTRs) as described previously. For short coding VNTRs analysis, we define polymorphic VNTRs as the VNTRs that have individuals with non-reference alleles ≥ 1% of the population.

To call small variants in long coding VNTRs, we set the minimum supporting reads threshold as 5. To find novel variants not reported by standard variant calling pipeline with short reads, we compared the variants found by code-adVNTR with the variants reported in VCF files.

#### Finding population-specific VNTR alleles

To investigate population-specific alleles in the short coding VNTRs, we calculated entropy for each VNTR. For each super population, we counted how many alleles (repeat count) were observed for each allele. Based on the allele count vector, we calculated normalized entropy $\frac{\sum_{i}p_{i}\;\ln\;p_{i}}{\ln\;n}$, where $p_{i}$ is the fraction of allele *i* and *n* is the total number of observed alleles of a VNTR.

#### MUC1 VNTR dataset

We used three positive samples that carries a cytosine frameshift variant in MUC1 VNTR and three negative samples identified from the previous study (Ekici et al., 2014). We also tested code-adVNTR on 271 DNA samples of German Chronic Kidney Disease cohort (Eckardt et al., 2012).

#### Generating alignment of reads

Code-adVNTR offers an option to generate alignment of supporting reads to the motif that has variants. We ran code-adVNTR with ‘-aln’ option to generate the alignments.

### Quantification and statistical analysis

To detect variants in coding VNTRs, we used likelihood ratio to perform an asymptotic chi-squared test following Wilks’s theorem. The significance level was set to 0.001. We applied the test on all candidate variants in 1000 Genomes Data. For the frameshift variants in MUC1 VNTR, we applied the same cutoff to call the variants.

## Data Availability

• This paper analyzes existing publically available data, with information in the key resources table. • All original code has been deposited at https://github.com/mehrdadbakhtiari/adVNTR/tree/enhanced_hmm and is publicly available. • Any additional information required to reanalyze the data reported in this paper is available from the lead contact upon request.

## Supplemental information

Document S1. Figures S1–S5

Table S1. Short coding VNTRs, related to STAR Methods

Table S2. Short coding polymorphic VNTRs, related to STAR Methods

Table S3. Long coding VNTRs, related to STAR Methods

Table S4. Novel small variants found in long coding VNTRs, related to STAR Methods
