# Supplementary material for: Detecting tandem repeat variants in coding regions using code-adVNTR
Source: iScience. 2022 Jul 19;25(8):104785. doi: 10.1016/j.isci.2022.104785 (PMC9379575; doi:10.1016/j.isci.2022.104785)
Supplement: Document S1. Figures S1–S5 [file mmc1.pdf]

**iScience, Volume *25***

## Supplemental information

# Detecting tandem repeat variants

# in coding regions using code-adVNTR

**Jonghun Park, Mehrdad Bakhtiari, Bernt Popp, Michael Wiesener, and Vineet Bafna**

## Supplemental Figures

**Figure S1. Comparison between single motif and multiple motif HMM, Related to Figure 1.**

An example of a tandem repeat with multiple motifs. When a read is sequenced from the tandem repeat region, single motif HMM would match the variations within motifs with mismatch/insertion/deletion states.

**Figure S2. The HMM for VNTRs, Related to Figure 1.**

The HMM is composed of three profile HMMs: prefix and suffix profile HMMs to align the flanking regions, and motif profile HMM to align multiple and partial number of motifs. Dotted lines connected to Start and End states indicate the transitions for reads that do not span the entire VNTR region.

**Figure S3. IGV screenshots of long read mapping on genomic sample NA12878 showing the small indels detected by code-adVNTR, Related to Figure 2.**

Long read validation of three single base heterozygous deletions identified at 2 VNTR loci in ZNF662 and ZNF717 by code-adVNTR using short read.

**Figure S4. Comparison of two groups of VNTRs that passed and failed Hardy-Weinberg equilibrium test, Related to Figure 3.**

(A) Distribution of VNTR lengths and (B) similarity scores of VNTRs that passed and failed Hardy–Weinberg equilibrium (HWE) test. The similarity score shows the highest number of matched nucleotides between a motif and a flanking sequence normalized by the length of the motif. VNTRs that have similar pattern with flanking regions are challenging because of the ambiguous boundary.

**Figure S5. Novel variants detected by code-adVNTR in 4 long coding VNTRs, Related to Figure 3.**

Novel variants detected by code-adVNTR within VNTRs in (A) *NEFH*, (B) *FMN2*, (C) *KRT-9*, and (D) *RP1L1* gene.

## Supplemental Tables

Table S1. Short coding VNTRs, Related to STAR Methods.

Table S2. Short coding polymorphic VNTRs, Related to STAR Methods.

Table S3. Long coding VNTRs, Related to STAR Methods.

Table S4. Novel small variants found in long coding VNTRs, Related to STAR Methods.

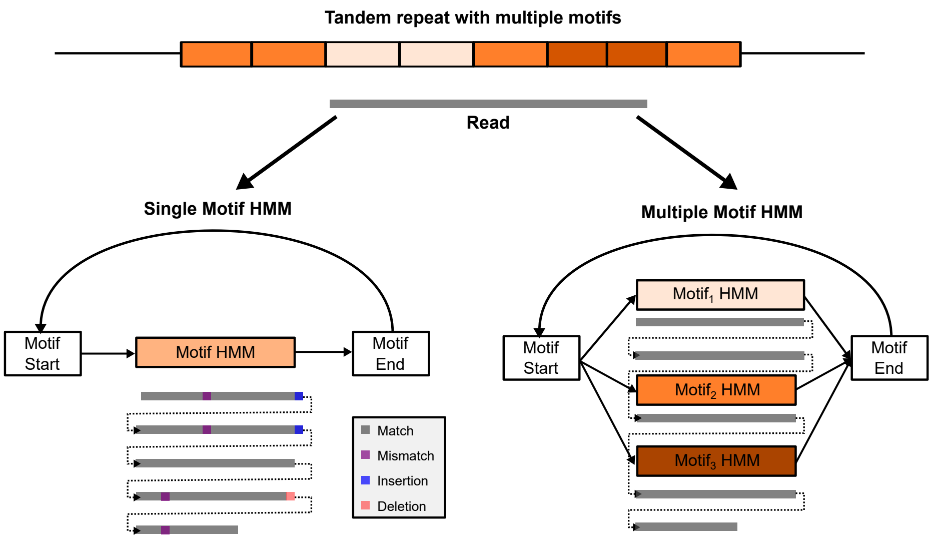


Figure S1: **Comparison between single motif and multiple motif HMM.** An example of a tandem repeat with multiple motifs. When a read is sequenced from the tandem repeat region, single motif HMM would match the variations within motifs with mismatch/insertion/deletion states.

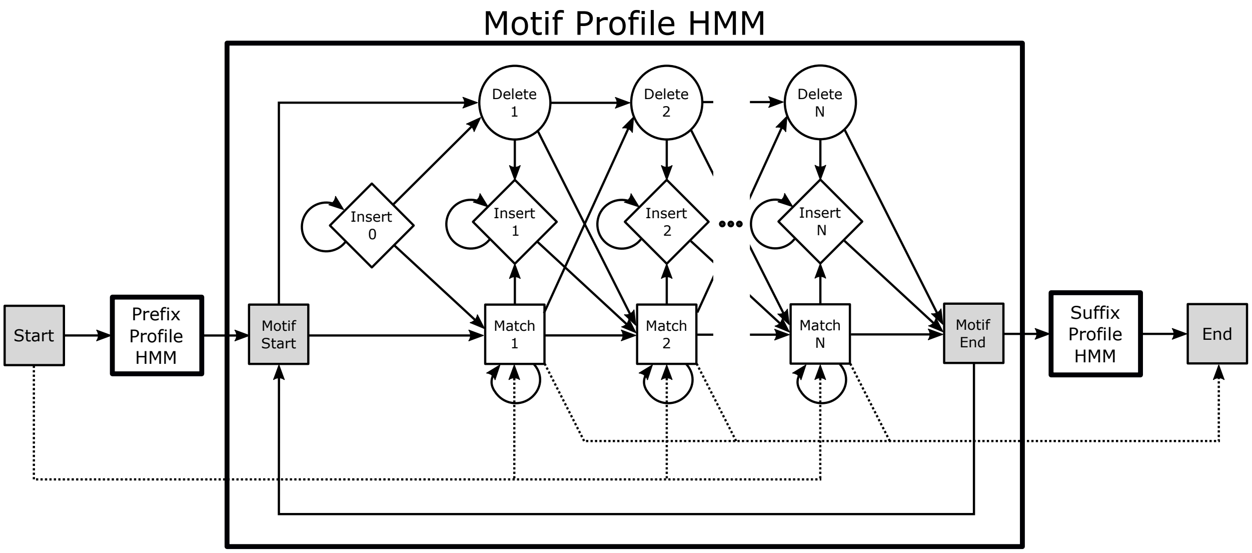


Figure S2: **The HMM for VNTRs.** The HMM is composed of three profile HMMs: prefix and suffix profile HMMs to align the flanking regions, and motif profile HMM to align multiple and partial number of motifs. Dotted lines connected to Start and End states indicate the transitions for reads that do not span the entire VNTR region.

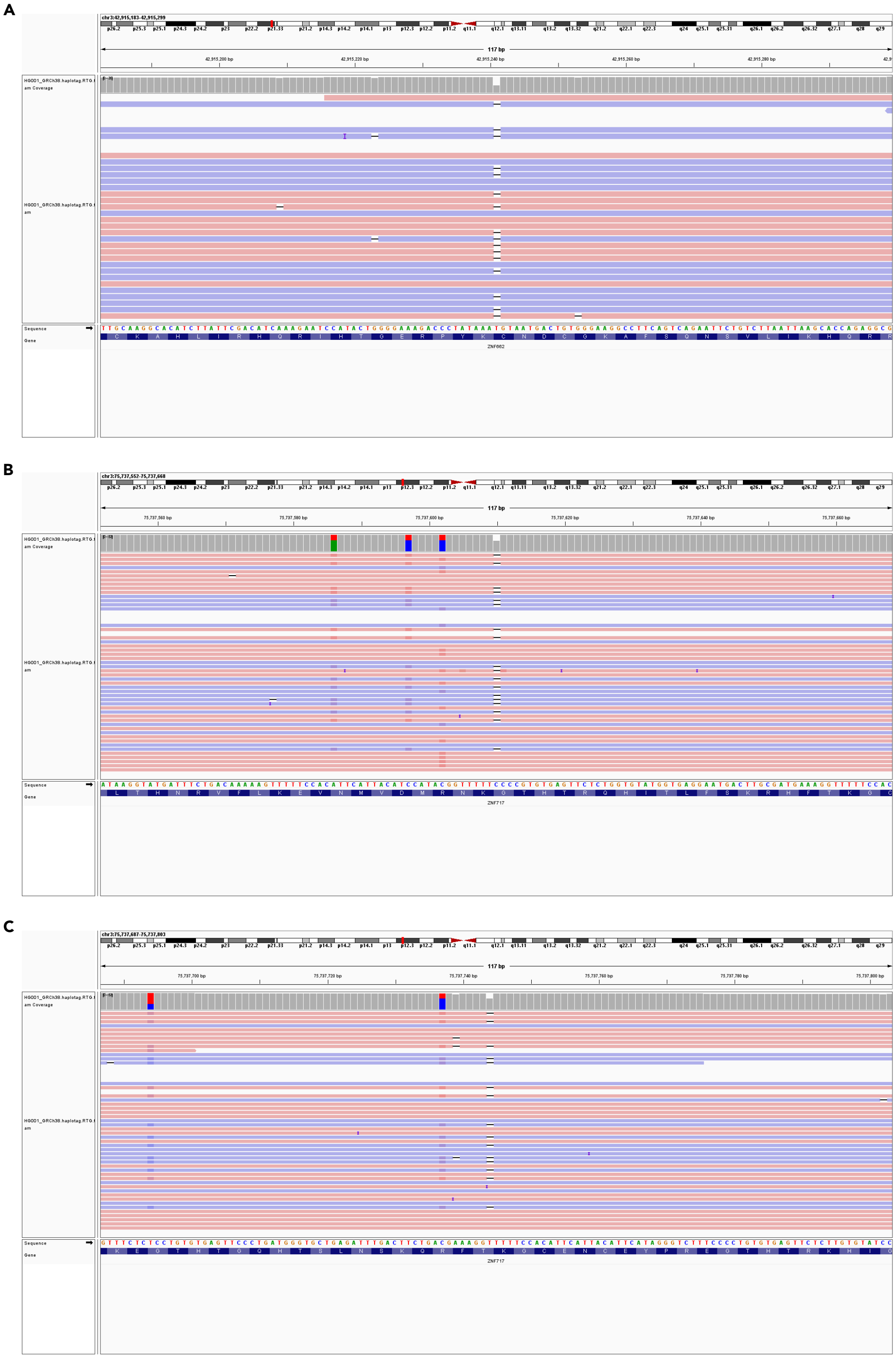


Figure S3: **IGV screenshots of long read mapping on genomic sample NA12878 showing the small indels detected by code-adVNTR.** Long read validation of three single base heterozygous deletions identified at 2 VNTR loci in ZNF662 and ZNF717 by code-adVNTR using short read.

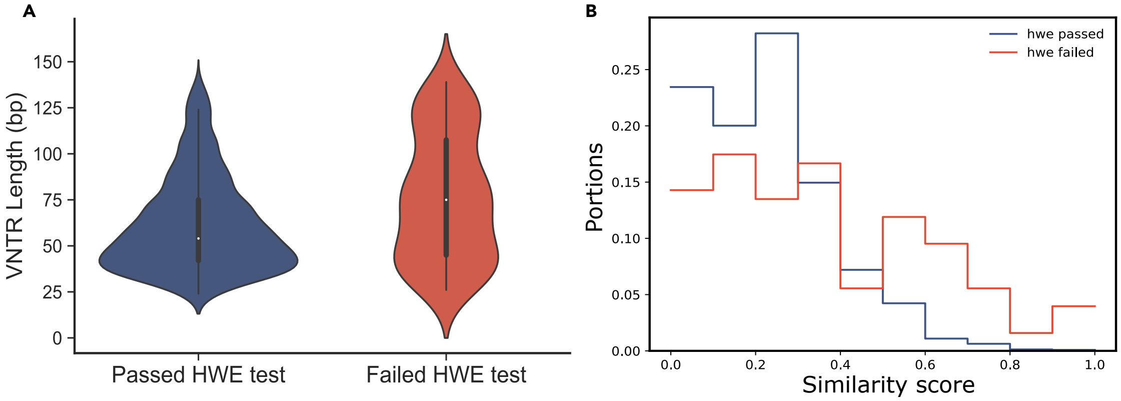


Figure S4: **Comparison of two groups of VNTRs that passed and failed Hardy-Weinberg equilibrium test.** (A) Distribution of VNTR lengths and (B) similarity scores of VNTRs that passed and failed Hardy–Weinberg equilibrium (HWE) test. The similarity score shows the highest number of matched nucleotides between a motif and a flanking sequence normalized by the length of the motif. VNTRs that have similar pattern with flanking regions are challenging because of the ambiguous boundary.

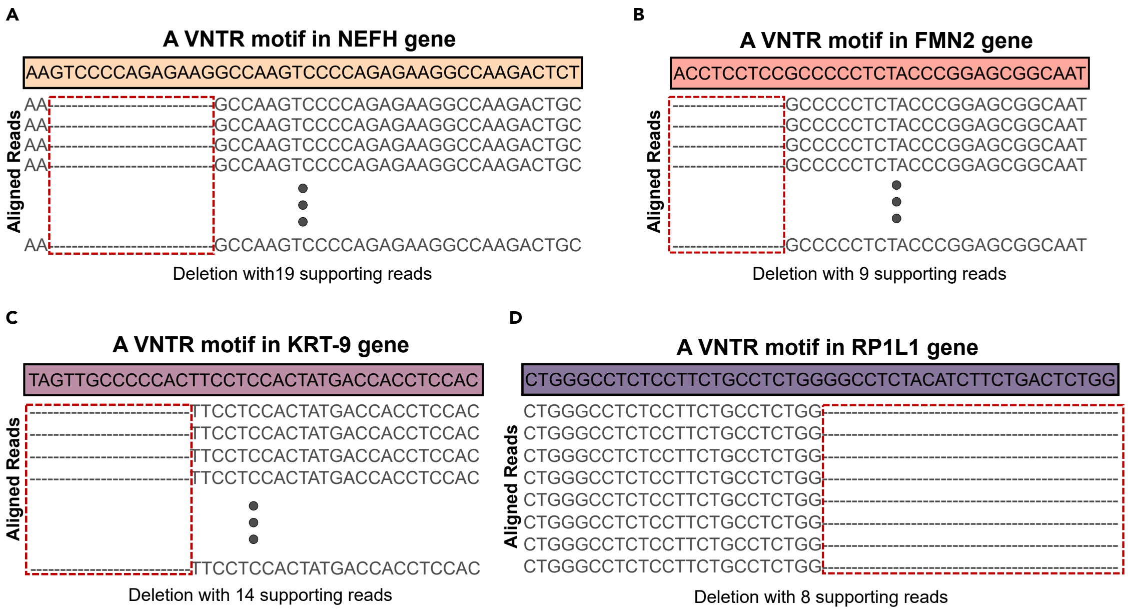


Figure S5: **Novel variants detected by code-adVNTR in 4 long coding VNTRs.** Novel variants detected by code-adVNTR within VNTRs in (A) *NEFH*, (B) *FMN2*, (C) *KRT-9*, and (D) *RP1L1* gene.
